# Genotype distribution of high-risk and potential high-risk HPV types among women in different screening and clinic settings in Ghana and Kenya

**DOI:** 10.1371/journal.pone.0355868

**Published:** 2026-08-28

**Authors:** Eliza Mari Kwesi-Maliepaard, Judith A. Osea-Larbi, Celestine K. Nyamari, Yakubu Alhassan, Adelaide K. Sromani, Vera M. Kotey, Stephanie Darko, Randy Tackie, Ruth Kiome, Aisha M. Mohammed, Erica Buadii, Emmanuel K. Quaye, Harry Akligoh, Barikisu A. Ibrahim, Patricia Kaba, Susan Florence Amoako, Esmy Kotey, Seth Agyemang, Joyce M. Ngoi, Lily Paemka, Kwaku Darkwa, Thywill Degley, Nathan Edward Siebu, Charles Ochieng’ Olwal, Collins M. Morang’a, Aida Manu, David Hutchful, Emmanuella Amoako, Frank G. Onyambu, Yaw Bediako

**Affiliations:** 1 Yemaachi Biotech Ltd, Accra, Ghana; 2 West African Genetic Medicine Centre, University of Ghana, Accra, Ghana; 3 Department of Biostatistics, School of Public Health, University of Ghana, Accra, Ghana; 4 West African Centre for Cell Biology of Infectious Pathogens, University of Ghana, Accra, Ghana; 5 Biochemistry, Cell and Molecular Biology Dept, University of Ghana, Accra, Ghana; 6 Sanford World Clinic, Mankessim, Ghana; 7 Ashesi University, Berekuso, Ghana; Sefako Makgatho Health Sciences University, SOUTH AFRICA

## Abstract

**Background:**

The burden of cervical cancer is highest in Africa. Human papillomavirus (HPV) infection is the major cause of cervical cancer. There are over 200 different HPV genotypes, with 15 considered (potential) high-risk genotypes for developing cancer. Due to a lack of systematic screening programs in most African countries, there is limited data available on the most prevalent HPV genotypes in the population. This study aimed to assess the prevalence and distribution of HPV genotypes in cohorts in Ghana and Kenya.

**Methods:**

We analyzed HPV genotyping data from cervical samples submitted for diagnostic HPV genotype testing between July 2022 and March 2025. Our database contained results from samples obtained from outreach screening events in Accra, the capital of Ghana (n = 680), a research project in Ghana outside of Accra (n = 426), clinician-collected samples submitted through clinics in Ghana (n = 118), samples from participants in Ghana who independently sought HPV genotyping through community-based providers, pharmacies, clinics, or online testing services outside organized screening programmes (n = 181) and samples from an outreach screening event in Kenya (n = 46). All samples were from women 18 years old and above. Positivity for HPV genotypes (HPV-16, 18, 31, 33, 35, 39, 45, 51, 52, 53, 56, 58, 59, 66, 68) was determined by RT-PCR, using the AmpFire HPV High Risk Genotyping kit.

**Results:**

HPV positivity in the Ghana screening group was 34.9%, with multiple genotypes detected in 39.2% of the positive cases. The main HPV genotype in the Ghana screening group was HPV-39 (19.0%), which was especially prevalent in the younger age group (<30 years), followed by HPV-53 (15.2%) and HPV-68 (15.2%). HPV infection (Odds Ratio (OR) 3.09, Confidence Interval (CI) 1.92–4.97, p < 0.001) and infection with multiple genotypes (OR 3.45, CI 1.42–8.38, p = 0.006) was associated with younger age in the Ghana screening group. In the Ghana research group HPV-53 was the most common genotype (18.9%), followed by HPV-39 (18.0%). In the clinician-collected group HPV-16 (18.6%) was the most common genotype. In the self-collected samples from participants who independently sought HPV testing HPV-39 (17.9%) and HPV-51 (17.9%) were the most common genotypes. In the much smaller Kenya screening group HPV-51 (46.2%) was the most prevalent genotype.

**Conclusions:**

Our study shows HPV genotype distribution in different settings. This emphasizes the importance of systematic population-wide HPV screening to understand local HPV infections patterns.

## Introduction

Cervical cancer is the fourth most common cancer in women globally [1]. African women are affected disproportionally, with the highest incidence and mortality rates for cervical cancer reported in Africa [1]. Of the top 20 countries with the highest cervical cancer burden in 2022, 18 are in Africa [2]. In Kenya, cervical cancer is the second most common cancer in women and has the highest mortality rate [3]. In Ghana, cervical cancer is also the second most common cancer in women and ranks third in mortality [3].

Infection with high-risk human papillomavirus (hr-HPV) is the main cause of cervical cancer. There are over 200 different HPV types reported, and new types are still being identified [4]. Fourteen HPV types (HPV-16, 18, 31, 33, 35, 39, 45, 51, 52, 56, 58, 59, 66 and 68) are considered carcinogenic and are referred to as high-risk genotypes for cervical cancer [5]. HPV-53 is considered a potential high-risk genotype [7]. HPV genotypes 6 and 11, which are found in 90% of genital warts, are considered low-risk [8]. Importantly, differences in the prevalence and distribution of high-risk genotypes between different populations have been reported [9,10]. High prevalence of HPV-35 infection has been reported in Africa [11–13] and in women of African descent living in the US [14]. Within the African continent, the prevalence of HPV infection and the distribution of hr-HPV vary [15].

HPV vaccination is an important factor in reducing HPV infection and the subsequent risk of cervical cancer. Different vaccines target different HPV genotypes. Approved vaccines are either bivalent (targeting HPV-16 and 18) or quadrivalent (targeting HPV-6, 11, 16 and 18). There is one nonavalent vaccine which targets HPV-6, 11, 16, 18, 31, 33, 45, 52 and 58 [16,17]. As of 2023, 134 countries had fully introduced HPV vaccination [18]. HPV vaccination with the quadrivalent vaccine has been rolled out in Kenya since October 2019 [19]. Ghana included HPV vaccination with the quadrivalent vaccine in its routine immunisation schedules in September 2025 [20,21]. Since the currently available vaccines only cover a subset of the known high-risk genotypes there is a need for broader vaccine types covering more HPV genotypes. This is especially relevant for African countries, where genotypes such as HPV-35, that are currently not covered by a vaccine, are more prevalent [22].

Ghana does currently not have a national cervical cancer screening programme [23]. As a result, there is no nationwide data on HPV prevalence. Most data on HPV prevalence in Ghana comes from research projects that offered HPV screening to a selected group of participants, often involving women visiting the obstetrics and gynaecology departments at a hospital [24–28]. The type of cervical cancer screening in Ghana is determined by many factors, including costs and availability of equipment [22]. The HPV detection methods in these studies were mostly PCR-based, with some studies only identifying HPV-16, HPV-18 and ‘other high-risk genotypes’, while other studies identified all 15 high-risk HPV genotypes. The results from these study vary, with HPV-52 [26,27], HPV-66 [24], or HPV-16 [28] being reported as the most prevalent in the general population. Studies identifying HPV in women with cervical cancer or cervical lesions identified HPV-16 as the most prevalent [24,29]. Taken together this shows the need for more information about distribution of high-risk HPV genotypes in different settings in Ghana.

In Kenya, the ‘National Cervical Cancer Elimination Action Plan – 2025-2030’ aims to scale up HPV testing coverage from 6% to 70% in 2030 [30]. Currently, most cervical cancer screening is based on Visual Inspection with Acetic Acid (VIA), which is less accurate than the recommended HPV testing [30]. Cervical cancer screening in Kenya mostly uses an opportunistic approach, offering screening to individuals who present at health clinics rather than offering systematic population-based screening [31]. Data describing distribution of HPV genotypes in the general population is therefore still limited.

In this study, we analysed the prevalence of (potential) high-risk HPV genotypes in cervical samples received for HPV testing in Ghana and Kenya. Samples from HPV screening events in Ghana and Kenya were self-collected. Samples from women in Ghana who purchased an HPV genotyping test through community-based providers, pharmacies, clinics, or online testing services outside organized screening events were also self-collected. Samples sent in through clinics in Ghana were clinician-collected. We show that prevalence and distribution vary between different demographic groups, highlighting the importance of nationwide screening to understand HPV infection patterns.

## Materials and methods

### Study type

This study is a cross-sectional study describing the secondary analysis of a database of HPV genotyping results. The HPV genotyping was performed as a diagnostic test on samples submitted through screening outreach events, from clinics, or from women who purchases an HPV test from pharmacies, clinics or online.

### Sample size

No prior sample size calculation was performed, as the HPV genotyping test was conducted as a diagnostic service rather than a formal research project. The final sample size was a convenience sample of all cervical swabs collected and processed from July 2022 till March 2025 at Yemaachi Biotech in Accra, Ghana. The majority of the samples (n = 680) were collected during outreach screening events in Accra. Another group of samples (n = 426) were processed and analyzed as part of the SWOGBACC research project [32]. The research samples were collected in Mankessim, in the Central Region of Ghana. The third group of samples were 118 cervical swabs obtained by a clinician. The clinics that sent in clinician-collected samples were Marie Stopes Ghana, Violet Medical Centre, and Elitecare Medical Centre, all located in Accra, Ghana. The next group (n = 181) were self-collected samples from participants who independently sought HPV genotyping through community-based providers, pharmacies, clinics, or online testing services outside organized screening programmes. The samples in this self-collected non-screening group were submitted from locations throughout Ghana. In Kenya, 46 samples were collected and processed at a January 2024 outreach screening event in Taita-Taveta county by Yemaachi Biotech in Kenya. The outreach screening events in Accra and Taita-Taveta county were held in public places (e.g., parks and malls) and offered free or discounted HPV testing for any interested women age 18 and above.

### Study design, data and sample collection

Participants obtained a Sheba HPV genotyping test [33], this is a branded HPV genotyping service from Yemaachi Biotech. The Sheba HPV genotyping test was offered for free or at reduced costs during screening outreach events, or purchased through a clinic, pharmacy or online. For the SWOGBACC study participants the Sheba HPV genotyping test was part of the study. All women whose results are included in the study were aged 18 years and above. At screening outreach events in Ghana and Kenya, cervical samples were self-collected using an Evalyn® Brush (Rovers Medical Devices) and submitted for diagnostic HPV-testing by the participants within 48 hours of taking the sample. Samples from the SWOGBACC research project were also self-collected using the Evalyn® brush and submitted for processing within 48 hours. Samples submitted through clinics were clinician-collected using a cotton swab and submitted for processing within 48 hours. The self-collected non-clinic samples sent in from participants who independently sought HPV genotyping through community-based providers, pharmacies, clinics, or online testing services outside organized screening programmes were also self-collected using the Evalyn® brush and submitted for processing within 48 hours. All samples were submitted for diagnostic HPV-testing within 48 hours of taking the sample. The participants provided their date of birth on the sample submission form. This was used the calculate the age of the participant on the day of sample reception. Upon receipt, samples were stored at room temperature and processed within three days. Samples were processed in the country where they were collected.

### HPV genotype identification by RT-PCR

Diagnostic HPV testing for the Sheba HPV genotyping test was performed using the AmpFire HPV High Risk Genotyping kit (Atila Biosystems) (v3.3 and v4.0) and was performed on all samples. This kit detects HPV genotypes 16, 18, 31, 33, 35, 39, 45, 51, 52, 53, 56, 58, 59, 66, and 68. Briefly, the Evalyn brush or the cotton swab was placed in a tube containing viral transport medium (Sanbao Gh Pharmaceuticals Ltd), and the cell suspension was stored at −20 °C for a maximum of 4 weeks. A 500 µl aliquot of the cell suspension was taken, spun at 14,000 rpm for 10 minutes, and the supernatant was discarded. The pellet was vortexed, and DNA was extracted by lysing the cells with 1x lysis buffer from the AmpFire kit at 95 °C for 10 min (v3.3) or 20 min (v4.0). The four different master mixes from the AmpFire kit were prepared by combining the reaction mix with the primer mix. From samples processed with the AmpFire kit v3.3, 2µl of extracted DNA was added to 23µl of master mix. For samples processed with v4.0 of the AmpFire kit, 5µl of extracted DNA was added to 20µl master mix. The PCR procedure was performed according to the manufacturer’s instructions, using a MA-6000 real-time quantitative thermal cycler (Sansure) in Ghana, or a CFX Bio-Rad PCR system in Kenya. An isothermal reaction was performed at 60°C, taking fluorescence readings at the FAM, HEX, CY5 and ROX channels once every minute for a total of 60 minutes. Results with a valid internal control signal were analyzed. Samples that did not pass the internal control were re-processed until a valid result was obtained. A total of three samples were not adequate to obtain results initially, in these cases results were obtained after resampling.

### Feedback of HPV genotyping results to the participants

Participants who participated in outreach screening events in Accra or who submitted a self-collected sample from a Sheba test, received their results via email. For those screened through the outreach screening event in Kenya, results were shared with a clinician from the Ministry of Health, who communicated them to the participants. Individuals who tested positive for hr-HPV were linked to appropriate care. The Ghana research study was conducted in the Sanford World Clinic – Mankessim and the clinical portion of the SWOGBACC study, including HPV testing, utilized the clinical operations. HPV results were returned to study participants through standard clinic test reporting procedures. Participants who submitted their samples through a clinic received their results through their doctor at the clinic. The results indicated whether one or more of the following HPV genotypes were detected (HPV16, 18, 31, 33, 35, 39, 45, 51, 52, 53, 56, 58, 59, 66, 68) and specified the specific genotypes detected. The report sent to the participants advised that ‘Females 30 years of age or older who have no abnormal cervical cytology but are high-risk HPV positive, especially for HPV16 and HPV18, should consult a doctor and request a vaginoscopy’.

### Data collection and processing

De-identified data was entered in VIAL, a web-based sample management system (Yemaachi Biotech). Data was retrieved in CSV format on 09/10/2025 and manually cross-checked for completeness. Each participant was assigned a unique identification number. Data contained: sex, age, and the results of the HPV genotyping test. Clinical data, including cytology, were not part of our database. The de-identified data were visualized using GraphPad Prism (v10.2.3). Stata MP version 18 was used for statistical analysis. The difference in age distribution between the different groups was assessed using a Bartlett’s equal-variances test. A Bonferroni-corrected pairwise comparison was used to compare the age distribution in each of the groups and the age distribution was shown using truncated violin plots. Clustered stacked bar charts were used to describe the study participants by HPV status and single versus multiple genotype infection. HPV-53 was classified as a potential high-risk genotype, all other genotypes included in the test (HPV-16, 18, 31, 33, 35, 39, 45, 51, 52, 56, 58, 59, 66, 68) were classified as high-risk genotypes. Multiple genotype infection was defined as any sample where two or more (potential) high-risk HPV genotypes were detected in the same sample. Co-occurrence of two HPV genotypes in a sample is described in a matrix depicting how often each combination of two different genotypes was found among the samples with multiple genotype infections. A bivariant association was used to examine HPV infection status and infection with individual genotypes in different age groups. A binary logistic regression model was used to estimate the odds ratios (ORs), 95% confidence intervals (CIs) and respective *p-*values for HPV positivity and infection with multiple genotypes among the Ghana screening group, with the different age groups as the predictive factor. A *p*-value less than 0.05 was considered statistically significant.

### Ethical approval

This study has been approved by the Noguchi Memorial Institute for Medical Research Institutional Review Board (NMIMR-IRB) in Accra, Ghana (NMIMR-IRB CPN 127/24–25) and by the Meru University Institutional Research & Ethics Review Committee (MIRERC) in Kenya (MIRERC 072/2025). The SWOGBACC study received IRB approval (00006220) from the Ethics and Protocol Review Committee of the College of Health Sciences, University of Ghana.

## Results

### Demographics

**Fig 1:**
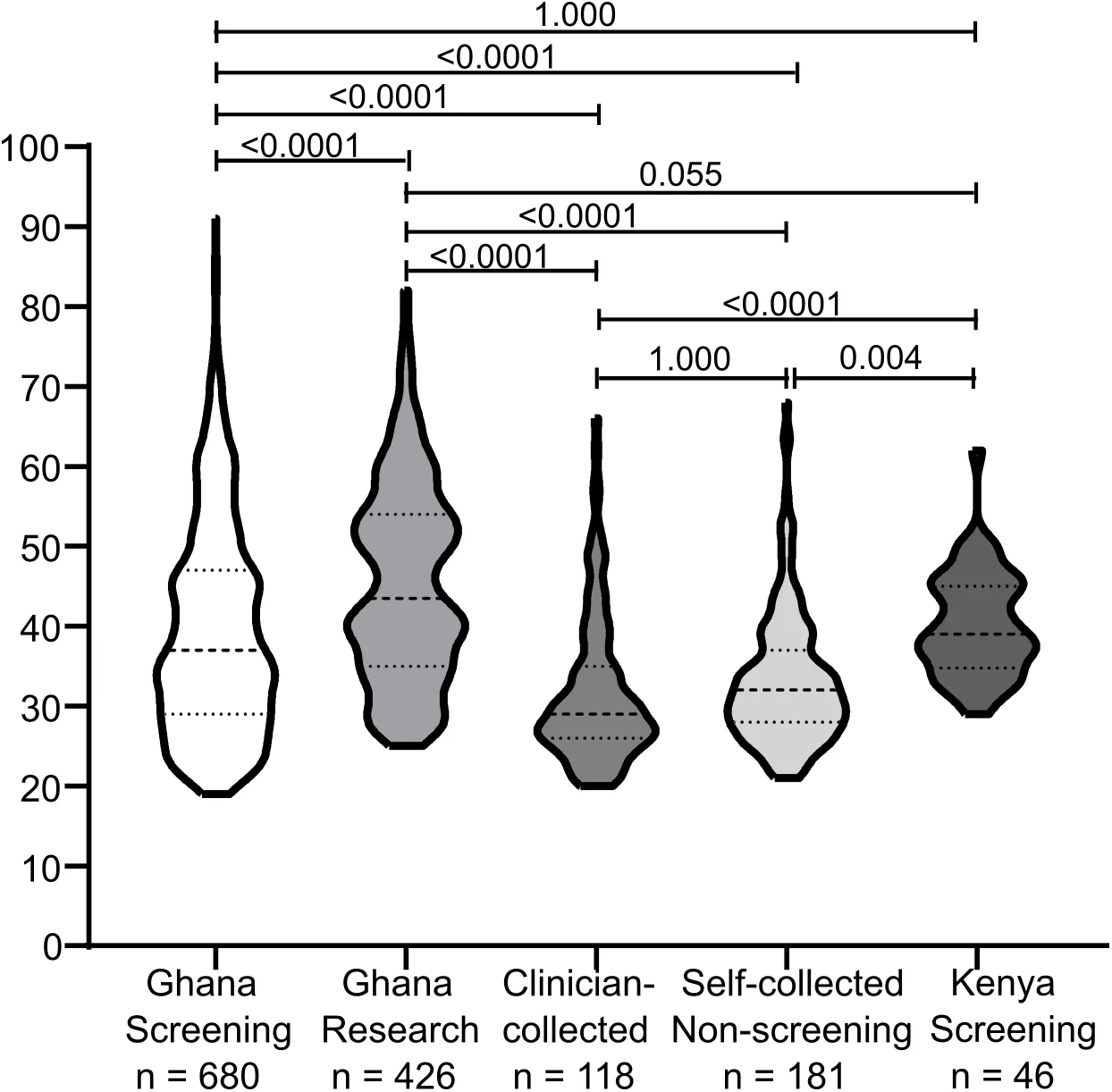
Age distribution. Age distribution in each of the study groups depicted in Violin plot. The violin plots are truncated to indicate the minimal and maximal ages of the participants. A Bonferroni corrected pair wise comparison was performed to assess age distribution between each of the groups.

The age of the participants in the screening group in Ghana (n = 680) ranged from 19 to 91, with a median age of 37 (IQR 29–47). Most participants were within the 30–39-year age group (Table 1). In the research group (n = 426) the age ranged from 25 to 82, with a median of 43.5 (IQR 35.25–53.75. The majority of the clinician-collected cervical swab samples were from participants in the age group 19–29 years with a median age of 29 (IQR 26–35). In the self-sampled non-screening group most participants were in the 30–39 year age bracket, with a median age of 32 (IQR 28–37). In the Kenya screening group the median age was 39 (IQR 35.25–45.0) with most participants in the 30–39 year age group. Analysis of the age distribution between the different age groups using a Bartlett’s equal-variances test showed that the age distribution was not the same across all groups (p < 0.0001) (Fig 1). A Bonferroni-corrected pairwise comparison showed that only between the Ghana Screening groups vs Kenya screening group, Ghana Research group vs Kenya group, and clinician-collected vs self-collected non-screening, the age distribution was not significantly different (Fig 1).

**Table 1:** Age distribution of the participants in the different groups.

Age group	Ghana screening n = 680 n (%)	Ghana Research n = 426 n (%)	Ghana clinic Swabs n = 118 n (%)	Self-collected non-screening n = 181 n (%)	Kenya Screening n = 46 n (%)
19-29	185 (27.2)	45 (10.6)	62 (52.5)	67 (37.0)	1 (2.2)
30-39	203 (29.9)	112 (26.3)	35 (29.7)	82 (45.3)	23 (50)
40-49	152 (22.4)	101 (23.7)	15 (12.7)	23 (12.7)	18 (39.1)
50-59	77 (11.3)	110 (25.8)	4 (3.4)	6 (3.3)	3 (6.5)
60 +	63 (9.3)	58 (13.6)	2 (1.7)	3 (1.7)	1 (2.2)

### HPV prevalence and genotype distribution in the Ghana screening group

**Fig 2:**
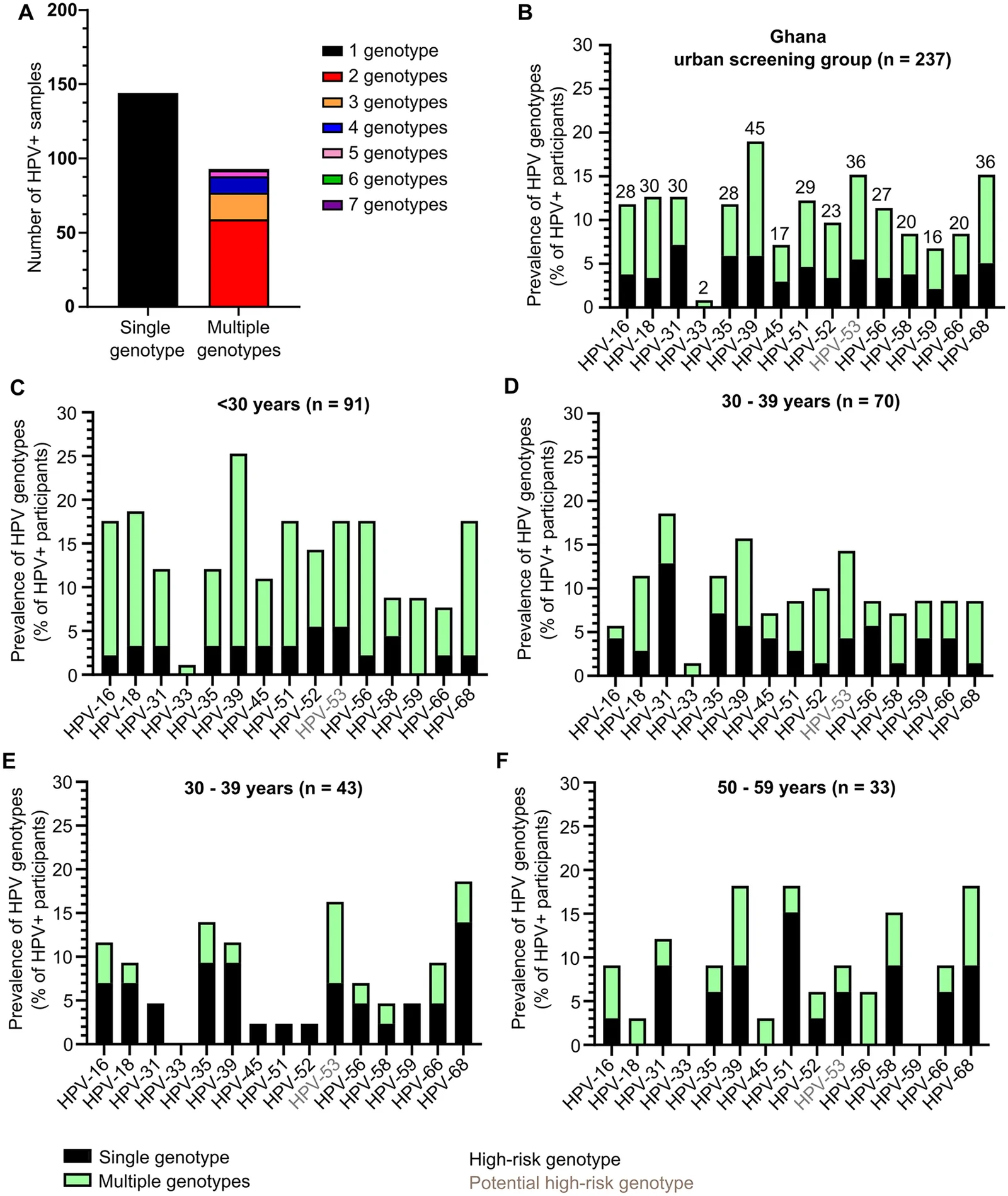
Distribution of HPV genotypes in the Ghana screening group. A) Number of HPV positive samples in the Ghana screening group, the number of HPV genotypes detected in one sample is indicated. B) The prevalence of HPV genotypes in the Ghana screening samples. C-F) Distribution of HPV genotypes per age group for the Ghana screening group. B-E) Prevalence is calculated using the number of HPV-positive participants as the denominator, the numbers above the bars indicate the absolute number of each genotype detected. Colours indicate prevalence of each genotype in a single genotype infection (black) or in a multiple genotype infection (green). High-risk genotypes are depicted in black; potential high-risk genotypes are depicted in grey.

HPV was detected in 237 (34.9%) of the 680 screening samples collected in Ghana. From these samples, multiple genotypes were detected in 93 (39.2%) cases, with a maximum of up to seven different genotypes detected in one sample (Fig 2A). The most common HPV genotypes detected in the screening samples from Ghana was HPV-39 (19.0%), followed by HPV-53 (15.2%), and HPV-68 (15.2%) (Fig 2B). The least common genotype was HPV-33 (0.8%), which was only found in infections with multiple genotypes (Fig 2B). Within the Ghana screening group, the patterns of genotypes differed between age groups. In the youngest population (<30 years), HPV-39 (25.3%) was the most prevalent genotype, followed by HPV-18 (18.7%) (Fig 2C). This is in contrast to the age group 30−39 years where HPV-31 (18.6%) was the most prevalent, followed by HPV-39 (15.7%) (Fig 2D). In the age group 40−49 years HPV-68 (18.6%) was the most detected genotype, followed by HPV-53 (16.3%) (Fig 2E). In the 50 + -year age group, HPV-51 (9.1%) and HPV-68 (9.1%) were the most common genotypes (Fig 2F). Table 2 provides an overview how often each HPV genotype co-occurred with another HPV genotype. The most frequently observed combination of genotypes was HPV-39 and HPV-68, which were found together in 12 samples, mostly in the < 30 years age group (n = 10). Other combinations often found together were HPV-53 & HPV-68 (n = 8), HPV-18 & HPV-39 (n = 7) and HPV-31 & HPV-39 (n = 7). This mostly reflects the high prevalence of these genotypes, especially in the younger age group. Together this data shows how HPV infection patterns differ between different age groups.

**Table 2:** The different HPV genotype combinations found in the Ghana Screening data.

	HPV-18	HPV-31	HPV-33	HPV-35	HPV-39	HPV-45	HPV-51	HPV-52	HPV-53	HPV-56	HPV-58	HPV-59	HPV-66	HPV-68
HPV-16	5	3	1	3	4	3	6	1	4	3	3	2	3	3
HPV-18		3	2	3	7	3	3	0	6	4	1	4	1	4
HPV-31			0	1	7	0	3	1	5	1	1	2	1	4
HPV-33				0	1	0	0	0	0	0	0	0	0	0
HPV-35					2	1	3	2	1	0	3	3	3	2
HPV-39						0	1	5	3	4	4	2	3	12
HPV-45							5	2	1	3	2	3	0	1
HPV-51								0	4	3	3	4	1	3
HPV-52									1	4	2	0	2	1
HPV-53										6	0	1	1	8
HPV-56											1	2	0	3
HPV-58												1	1	1
HPV-59													0	3
HPV-66														0

Total number of samples with multiple genotype infection: 93

Total number of HPV positive samples: 237

### Higher rates of HPV infection and multiple genotype infections in younger women in the Ghana screening group

Bivariate association showed a significant correlation (p < 0.001) between the different age groups and testing positive for HPV infection in the Ghana Screening group (Table 3). A similar association was reported for having multiple genotypes detected in the same sample (Table 3). A binary logistic regression model was used to estimate the odds of HPV positivity in the different age groups (Table 4). The odds of HPV positivity (OR 3.09, CI 1.92–4.97, p < 0.001) were higher in younger age groups compared to the eldest age group. Similarly, results from binary logistic regression among the HPV positive samples in the Ghana screening groups showed that the youngest age group was associated with higher odds of infection with multiple genotypes (OR 3.45, CI 1.42–8.38, p = 0.006) (Table 4).

**Table 3:** Bivariate association between age groups and HPV infection status, infection type and individual genotypes.

			Age group		
		Total	<30	30+	p-value
**Outcome**	**Status**	n = 680	n = 207	n = 473	
HPV infection status	HPV infection	237 (34.9%)	101 (48.8%)	136 (28.8%)	<0.001
	No infection	443 (65.1%)	106 (51.2%)	337 (71.2%)	
Infection type	Multiple	93 (39.2%)	53 (52.5%)	40 (29.4%)	<0.001
	Single	144 (60.8%)	48 (47.5%)	96 (70.6%)	
HPV16	No	209 (88.2%)	85 (84.2%)	124 (91.2%)	0.098
	Yes	28 (11.8%)	16 (15.8%)	12 (8.8%)	
HPV18	No	207 (87.3%)	84 (83.2%)	123 (90.4%)	0.096
	Yes	30 (12.7%)	17 (16.8%)	13 (9.6%)	
HPV31	No	207 (87.3%)	88 (87.1%)	119 (87.5%)	0.930
	Yes	30 (12.7%)	13 (12.9%)	17 (12.5%)	
HPV33	No	235 (99.2%)	100 (99.0%)	135 (99.3%)	0.830
	Yes	2 (0.8%)	1 (1.0%)	1 (0.7%)	
HPV35	No	209 (88.2%)	88 (87.1%)	121 (89.0%)	0.660
	Yes	28 (11.8%)	13 (12.9%)	15 (11.0%)	
HPV39	No	192 (81.0%)	78 (77.2%)	114 (83.8%)	0.200
	Yes	45 (19.0%)	23 (22.8%)	22 (16.2%)	
HPV45	No	220 (92.8%)	91 (90.1%)	129 (94.9%)	0.160
	Yes	17 (7.2%)	10 (9.9%)	7 (5.1%)	
HPV51	No	208 (87.8%)	83 (82.2%)	125 (91.9%)	0.024
	Yes	29 (12.2%)	18 (17.8%)	11 (8.1%)	
HPV52	No	214 (90.3%)	88 (87.1%)	126 (92.6%)	0.160
	Yes	23 (9.7%)	13 (12.9%)	10 (7.4%)	
HPV53	No	201 (84.8%)	81 (80.2%)	120 (88.2%)	0.088
	Yes	36 (15.2%)	20 (19.8%)	16 (11.8%)	
HPV56	No	210 (88.6%)	85 (84.2%)	125 (91.9%)	0.063
	Yes	27 (11.4%)	16 (15.8%)	11 (8.1%)	
HPV58	No	217 (91.6%)	93 (92.1%)	124 (91.2%)	0.800
	Yes	20 (8.4%)	8 (7.9%)	12 (8.8%)	
HPV59	No	221 (93.2%)	93 (92.1%)	128 (94.1%)	0.540
	Yes	16 (6.8%)	8 (7.9%)	8 (5.9%)	
HPV66	No	217 (91.6%)	93 (92.1%)	124 (91.2%)	0.800
	Yes	20 (8.4%)	8 (7.9%)	12 (8.8%)	
HPV68	No	201 (84.8%)	84 (83.2%)	117 (86.0%)	0.540
	Yes	36 (15.2%)	17 (16.8%)	19 (14.0%)	

**Table 4:** Binary logistic regression model of the association between age and HPV positivity and multiple genotype infection in the Ghana screening group.

	Odds of positive HPV test in the Ghana screening group			Odds of multiple genotypes among all Positive HPV tests in the Ghana screening group	
**Age group (years)**	**OR (95% CI)**	**P-value**		**OR (95% CI)**	**P-value**
<30	3.09 [1.92, 4.97]	<0.001		3.45 [1.42, 8.38]	0.006
30-39	1.61 [0.98, 2.65]	0.062		2.08 [0.81, 5.38]	0.130
40-49	1.28 [0.76, 2.16]	0.359		0.71 [0.24, 2.16]	0.551
50+	1.00 [reference]			1.00 [reference]	

OR: odds ratio. CI: confidence interval.

Further bivariate analysis showed that infection with HPV-51 was associated with the younger age group (<30 years) (p = 0.024). The other genotypes tested in this study did not correlate with the age group of the participants in the Ghana screening group (Table 3).

### HPV distribution in different screening and clinic settings in Ghana and Kenya

**Fig 3:**
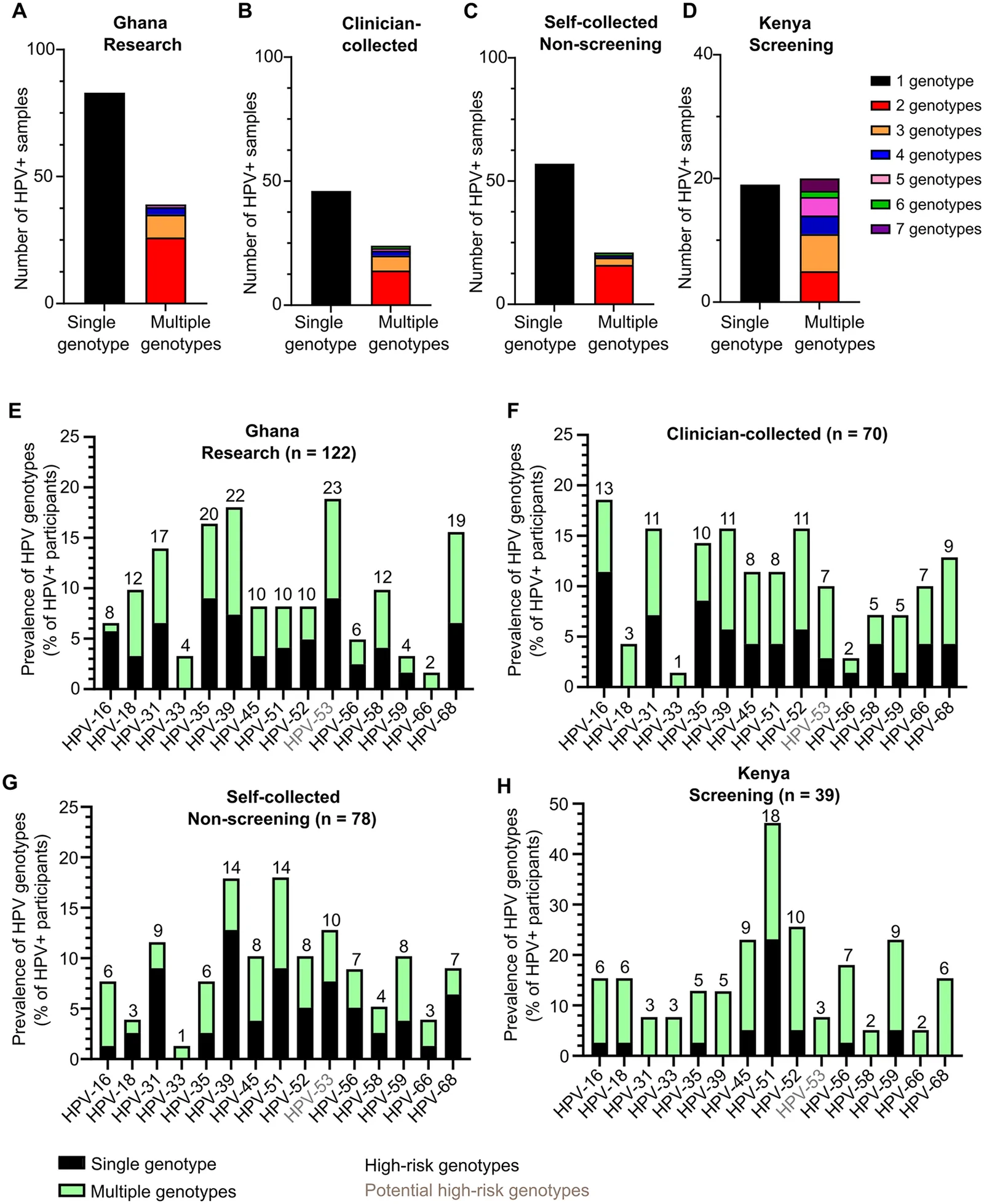
HPV genotype distribution in the Ghana clinic and Kenya screening group. A-D) Number of HPV positive samples in the Ghana research group (A), clinician-collected group (B), self-collected non-screening group (C) and in the Kenya screening group (D), the number of HPV genotypes detected in one sample is indicated. E) Distribution of HPV genotypes in the Ghana research group. F) Distribution of HPV genotypes in the clinician-collected group. G) Distribution of HPV genotypes in the self-collected non-screening group. H) Distribution of HPV genotypes in the Kenya screening group. E-H) Prevalence is calculated using the number of HPV-positive participants as the denominator, the numbers above the bars indicate the absolute number of each genotype detected. Colours indicate prevalence of each genotype in a single genotype infection (black) or in a multiple genotype infection (green). High-risk genotypes are depicted in black; potential high-risk genotypes are depicted in grey.

In the Ghana research group HPV positivity was detected in 28.6% (122/426), with multiple genotypes in 32.0% (39/122) of the positive samples (Fig 3A). In the clinician-collected samples sent in by the clinics in Ghana, the percentage of HPV positivity was 59.3% (70/118), with 24 (34.2%) samples having multiple genotypes (Fig 3B). In the self-collected non-screening samples the percentage of HPV positivity was 42.1% (78/181), with 26.9% (21/78) multiple genotype infections (Fig 3C). From the samples collected in Kenya, HPV was detected in 39 samples (84.8%). From these samples, 20 (51.3%) had multiple genotypes (Fig 3D).

In the Ghana research group HPV-53 was the most prevalent genotype, found in 18.9% of all positive samples, followed by HPV-39 (18.0%) and HPV-68 (15.6%) (Fig 3E). The least common genotypes in the Ghana research group were HPV-66 (1.6%), HPV-59 (3.3%) and HPV-33 (3.35%), with HPV-66 and HPV-33 only found in infections with multiple genotypes (Fig 3E). In the clinician-collected samples, the most common genotypes were HPV-16 (18.6%) followed by HPV-31, HPV-39, and HPV-52 (all 15.7%) (Fig 3F). The least common genotypes in the clinician-collected samples were HPV-33 (1.4%), HPV-56 (2.9%) and HPV-18 (4.3%), with HPV-33 and HPV-18 only present in infections with multiple genotypes (Fig 3F). In the self-collected non-screening samples the most common genotypes were HPV-39 (17.9%) and HPV-51 (17.9%), followed by HPV-53 (12.8%) (Fig 3G). In this group HPV-33 was the least common genotype (1.3%) and only found in infection with multiple genotypes (Fig 3G). In the samples from Kenya, HPV-51 (46.2%) was the most common genotype, and HPV-58 (5.1%) and HPV-66 (5.1%) were the least common (Fig 3H).

### HPV-vaccine coverage of the HPV genotypes detected

Current HPV vaccines cover up to 9 different HPV genotypes (HPV-6, 11, 16, 18, 31, 33, 45, 52 and 58). In this study, cervical samples were tested for 15 different high-risk and potential high-risk genotypes. In the Ghana screening group, those genotypes were detected 387 times in total, with 150 (38.8%) being vaccine genotypes (Table 5). Within the different age groups 30.0% (40−49 years), 35.6% (50 + years), 39.2% (<30 years) and 43.9% (30−39 years) of genotypes identified were vaccine genotypes (Table 5). In the Ghana research group 43.7% (73/167) were vaccine genotypes. From the clinician-collected samples, 45.6% (52/114) were vaccine genotypes. In the self-collected non-screening samples vaccine genotypes were 36.1% (39/108) of the total HPV genotypes observed. Among the Kenya samples, 41.5% (39/94) of the genotypes detected would be covered by the nonavalent vaccine (Table 5). HPV-16 and 18, the high-risk genotypes covered by the bivalent and quadrivalent vaccines, account for 15.0% (58/387) in the Ghana screening group, 12% (20/167) in the Ghana research group, 14.0% (16/114) in the clinician-collected samples, 8.3% (9/108) in the self-collected non-screening samples and 12.8% (12/94) in the Kenya samples (Table 5).

**Table 5:** HPV vaccine coverage of the hr-HPV genotypes detected in the three cohorts.

	hr-HPV genotypes covered by the Bivalent/Quadrivalent Vaccine n (%)	hr-HPV genotypes covered by the Nonavalent Vaccine n (%)
Ghana screening group (n = 387 HPV genotypes in total)	58 (15.0)	150 (38.8)
<30 years (n = 189 HPV genotypes)	33 (17.5)	76 (40.2)
30–39 years (n = 102 HPV genotypes)	12 (11.8)	43 (42.2)
40–49 years (n = 51 HPV genotypes)	9 (17.6)	15 (29.4)
50 + years (n = 45 HPV genotypes)	4 (8.9)	16 (35.6)
Ghana research group (n = 179 HPV genotypes in total)	20 (11.2)	73 (40.8)
Clinician-collected (n = 111 HPV genotypes in total)	16 (14.4)	52 (46.8)
Self-collected non-screening (n = 108 HPV genotypes in total)	9 (8.3)	39 (36.1)
Kenya group (n = 94 HPV genotypes in total)	12 (12.8%)	39 (41.5)

## Discussion

The prevalence of different HPV genotypes differs between geographical regions and demographics. This study aimed to assess the prevalence and distribution of HPV genotypes in Ghanaian and Kenyan cohorts. Sub-Saharan Africa is disproportionally burdened by cervical cancer, but data on the prevalence of high-risk and potential high-risk genotypes in the general population are limited. Here we show the distribution of (potential) high-risk HPV genotypes in women in different screening and clinic settings in the two countries, Ghana and Kenya.

Prevalence of HPV positivity among Ghanaian women has been reported to be between 10.7% and 45.8% [24,25,27,34]. This is in line with the 34.9% prevalence that we observed in the Ghana screening group and the 28.6% in the Ghana research group in the current study. The HPV prevalence data from the clinician-collected (59.3%) and self-collected non-screening (42.1%) groups and the Kenya group (84.8%) should be interpreted carefully, since the number of samples in these groups is small. Direct comparison between the screening and clinic groups is not possible due to difference in age distribution, and the fact that participants in the outreach screening events got a free or discounted HPV genotyping test, whereas participants in the clinic group had to purchase the test. Interestingly, women who purchased an HPV genotyping test through a clinic, pharmacy or online were often young women. Furthermore, the difference in sample size between the Kenya and Ghana screening groups does not allow for direct comparison of the two groups. It should also be noted that the prevalence of HPV positivity varies across different studies and study sites [35]. These differences may reflect true biological differences, but should be interpreted cautiously since differences in population demographics, HPV testing and sampling methods can influence the results [36].

In the Ghana screening and research groups, we observed a high prevalence of HPV-39 (19.0% and 18.0% respectively). This is interesting, given that other studies in Ghana reported HPV-52, HPV-66 or HPV-16 as the most prevalent genotype [9,24,26–28]. Meta-analysis on HPV prevalence in sub-Saharan Africa reported HPV-16 and HPV-35 to be the most prevalent genotypes in West Africa [15]. A potential explanation for the differences in genotype distribution between our study and other studies may lie in the demographics of the participants. We observed the strongest dominance of HPV-39 in the youngest age group. Other studies used different age groups or did not include participants in the youngest age groups.

In the Ghana clinician-collected samples, HPV-16 (18.8%) was the most prevalent genotype. This is in line with HPV-16 being the major cause for cervical cancer globally and in Africa [9,10,12]. HPV-18, the other high-risk genotype in the bivalent and quadrivalent vaccines, had a much lower prevalence in the clinic samples. A lower prevalence of HPV-18 has been observed globally [37,38]. However, in West-Africa a higher HPV-18 prevalence has been reported, with HPV-18-lineage-C as an African specific lineage [39].

The most prevalent HPV genotypes that we detected in the samples collected in Kenya are HPV-51 (46.2%), followed by HPV-52 (25.6%), HPV-45 (23.1%) and HPV-59 (23.1%). A meta-analysis has reported HPV-16 and HPV-52 to be the most prevalent genotypes in East Africa. Another recent study from Kenya reports HPV-52 as the most common, followed by HPV-67 and HPV-16 [40]. It should be noted that the data from the Kenya group is based on a small number of participants and should therefore be interpreted with caution as it is not representative of the overall situation in Kenya.

In Sub-Saharan Africa, increased prevalence of HPV-35 in invasive cervical carcinoma and in precancerous lesions has been reported [10,12,13]. A similar link between HPV-35 and cervical carcinogenesis has been found in women of African ancestry living in the US [14]. Globally, HPV-35 accounts for 2% of invasive cervical cancer [10], in some African countries HPV-35 has been detected in more than 10% of cervical cancer cases [11,12]. In our Ghana clinician-collected clinic samples HPV-35 was detected in 10 out of the 70 HPV positive participants (14.3%). Due to limitations in the study design, we were unable to determine whether patients in whom HPV-35 was detected had normal cervical findings, precancerous lesions, or malignant disease.

From the 15 HPV genotypes tested in this study, HPV-53 was the only potential high-risk genotype in this study, the other 14 genotypes that were tested are confirmed high-risk [6]. HPV-53 was among the most prevalent genotypes in the Ghana screening group, the Ghana research group and the self-collected non-screening group. This is in line with HPV-53 infection being relative common globally [41]. The pathogenicity of a single HPV-53 infection has been reported as low [7,42], However, studies in women living with HIV (WLWH) in Kenya have shown a correlation between HPV-53 infection and reduced CD4 count [43] and reported HPV-53 as a stand-alone infection in invasive cervical cancer in a woman living with HIV in Kenya [44]. Another study from Kenya has shown that HPV-53 infection was associated with low-grade squamous intraepithelial lesions (LSIL) in WLWH [45]. HPV-53 is also among the genotypes with increased prevalence in WLWH in Ghana [46]. Inclusion of HPV-53 in screening tests in countries with a high HIV burden would therefore be advisable.

We observed higher rates of HPV positivity and of infections with multiple HPV genotypes in younger women (<30) compared to older women. This has been reported in multiple studies from different geographic locations [9,28,41,47]. It is thought that a combination of sexual behaviour and not having acquired immunity against HPV infection yet contributes to the increased rate of HPV infection in younger women [47]. Given the nature of our study, it was not possible to distinguish between incident infections, which will be cleared by the immune system within a couple of months, and persistent infections, which are not cleared by the immune system and can eventually progress to cancer, in the different age groups [48]. Although it has been shown that younger women have often incident infections, not all infections in this age group are cleared [47,49]. Further research into the duration and clearance of HPV infections, especially in younger women, is needed to optimize vaccination strategies.

Several studies have shown that HPV vaccination in Ghana and Kenya is cost-effective [19,50,51]. The costs differ per vaccine type, with a higher price for vaccine types that cover more HPV genotypes [19]. However, HPV-16 and HPV-18, the two high-risk genotypes covered in the bivalent and quadrivalent vaccines, only account for 8.4%−15% of all the genotypes detected in the different groups in this study. When more genotypes are includes, as is the case for the nonavalent vaccine, the coverage increases to 36.1% − 45.6%. This means that more than half of the (potential) high-risk HPV genotypes detected in these samples are not covered by any currently available vaccine. A recent study from Ghana reported HPV-59 and HPV-35, both not included in the current vaccines, as the most prevalent genotypes detected in cervical cancer [29]. High prevalence of non-vaccine HPV genotypes in Sub-Saharan Africa has been reported by others as well [11,15]. This warrants the development of novel HPV vaccines with a broader coverage, tailored to local infection patterns [22].

Samples obtained through screening events in Ghana and Kenya were collected through self-sampling. The same applies to samples from women who purchased an HPV genotyping test through a clinic, pharmacy or online. Samples submitted through clinics were clinician-collected. Unfortunately, we do not know the reason why an HPV genotyping test was purchased. It might be that self-collected samples are more often obtained for preventive HPV testing, whereas clinician-collected samples might come from women with symptoms warranting HPV testing. Self-sampling has been shown to be a reliable method of collecting cervical samples and has a high acceptance rate among participants [52,53]. The accuracy of HPV genotyping in self-collected samples versus clinician-collected samples has been reported to vary between studies [53–55]. While most studies report acceptable specificity and sensitivity of HPV testing in self-collected samples, there is wide range of accuracy [53]. This highlights the importance of using standardized sampling devices, transportation and processing. Our results do not allow for a direct comparison between self-collected and clinician-collected samples, since the settings in which these samples are collected differ and clinical data is lacking. Further research is needed to investigate the optimal sample collection strategy for HPV screening in Ghana and Kenya.

### Limitations

The size of the Kenya group in our study is small, and all samples were obtained from one outreach event in a rural location. Results should therefore be interpreted with caution and cannot be immediately extrapolated to describe national levels of HPV prevalence and distribution in Kenya. The samples from the Ghana screening group were mainly collected in Accra and may also not reflect nationwide patterns of HPV prevalence and distribution. It should also be noted that the sample types differed between different settings, with self-collected samples in the screening and research groups and both clinician-collected and self-collected samples in the clinic groups.

This study describes the analysis of data from a database containing HPV genotyping results. The demographic information available in the database is limited to age. Other socio-demographic characteristics that would be interesting for further analysis are not available. This also applies to other potentially interesting information on HIV status and sexually transmittable diseases. In addition, there was no data available on cervical cytology to determine the genotypes associated with cervical dysplasia. Further research is needed to investigate which of the high-risk HPV genotypes are most prevalent in cervical cancer patients in Ghana.

## Conclusion

In this study we analyzed a database of HPV genotyping data from women in Ghana and Kenya. Our study describes the distribution of HPV genotypes in different settings, with differences in HPV infection patterns observed among different age groups. We also show that more than 50% of the (potential) high risk genotypes found in our study groups are not covered by the currently available vaccines. This supports the need for further development of HPV vaccines tailored towards specific regional HPV genotypes distribution patterns.

### Recommendations

Our study highlights the importance of systematic population-wide HPV screening to monitor HPV infection patterns in different populations.

## Supporting information

S1 File Full dataset. The full dataset supporting the conclusions in this paper.
(XLSX)
